# Understanding caregivers' decision to vaccinate childhood cancer survivors against COVID‐19

**DOI:** 10.1002/cam4.6675

**Published:** 2023-11-08

**Authors:** Anica Ilic, Regine Haardoerfer, Gisela Michel, Cam Escoffery, Ann C. Mertens, Jordan Gilleland Marchak

**Affiliations:** ^1^ Faculty of Health Sciences and Medicine University of Lucerne Lucerne Switzerland; ^2^ Department of Pediatrics Emory University School of Medicine Atlanta Georgia USA; ^3^ Aflac Cancer & Blood Disorders Center Children's Healthcare of Atlanta Atlanta Georgia USA; ^4^ Rollins School of Public Health Emory University Atlanta Georgia USA

**Keywords:** caregivers, COVID‐19, health literacy, intention, psychological theory, survivors of childhood cancer, vaccines

## Abstract

**Background:**

Vaccination against COVID‐19 is recommended for childhood cancer survivors (CCS). This study aimed to identify antecedents contributing to caregivers' decisions to vaccinate CCS aged 5–17 years against COVID‐19 by applying the Theory of Planned Behavior.

**Methods:**

Participants in this cross‐sectional study completed an online survey assessing caregiver attitudes, subjective norms, perceived behavioral control, intention to vaccinate CCS, CCS vaccination status, COVID‐19 health literacy, and frequency of COVID‐19 information‐seeking. Surveys were completed between May and June 2022 following approval for the emergency use of COVID‐19 vaccines among children aged ≥5 years in the U.S. Data were analyzed using unadjusted linear regressions and structural equation modeling.

**Results:**

Participants were caregivers (*n* = 160, 87.5% biological mothers, 75.6% white/non‐Hispanic) of CCS (*n* = 160, 44.4% female, mean (*M*) = 12.5 years old, *M* = 8.0 years off treatment). 70.0% (*n* = 112) of caregivers and 53.8% (*n* = 86) of CCS received a COVID‐19 vaccine. Over one‐third (37.5%) of caregivers reported disagreement or indecision about future COVID‐19 vaccination for the CCS. Caregivers' intention (*β* = 0.962; standard error [S.E.] = 0.028; *p* < 0.001) was highly related to CCS vaccination status. Attitudes (*β* = 0.568; S.E. = 0.078; *p* < 0.001) and subjective norms (*β* = 0.322; S.E. = 0.062; *p* < 0.001) were associated with intention. Higher frequency of COVID‐19 information‐seeking (*β* = 0.313; S.E. = 0.063; *p* < 0.001) and COVID‐19 health literacy (*β* = 0.234; S.E. = 0.059; *p* < 0.001) had a positive indirect effect on intention through attitudes and subjective norms.

**Conclusions:**

Caregivers' vaccination intentions for minor CCS are highly related to vaccination behavior and shaped by attitudes, subjective norms, COVID‐19 health literacy, and frequency of COVID‐19 information‐seeking. Promoting tailored communication with caregivers of CCS and encouraging them to review reputable sources of information can address their vaccine hesitancy.

## 1 INTRODUCTION

Childhood cancer survivors (CCS) may be at higher risk of contracting coronavirus disease 2019 (COVID‐19),^1^ while CCS experiencing specific late effects of treatment face increased chances of a severe course of COVID‐19.^2^ These include chronic conditions such as gastrointestinal^3^ and endocrine problems^4^, ^5^ that might be risk factors for severe COVID‐19 in children.^6^ Furthermore, while a cancer history may be a risk factor for long‐lasting symptoms following COVID‐19 in adults,^7^ its long‐term impact on young CCS remains unknown. Given these risks, the ongoing prevalence of COVID‐19, and the continuing emergence of new variants,^8^ the Children's Oncology Group (COG) recommends that all eligible CCS receive a COVID‐19 vaccine.^9^ When survivors are minors, their caregivers decide if they receive the COVID‐19 vaccine; thus, understanding caregiver COVID‐19 vaccine decision‐making is essential to long‐term health outcomes for CCS.

Previous research indicates that parents of CCS were more likely to accept COVID‐19 vaccines for their CCS when they were confident in the governmental response to COVID‐19, received information on COVID‐19 from cancer care professionals, trusted in science, medicine, and vaccination, including COVID‐19 vaccinations, and believed that CCS are at greater risk of complications due to COVID‐19.^10^ Characteristics deterring their decision included concerns about the rapidness of vaccine development and its safety for children and CCS.^10^ A study addressing COVID‐19 vaccine hesitancy in parents of children with cancer and CCS found greater hesitancy was associated with both parents' factors (younger age, male sex, lower income) and children's factors (younger age at diagnosis and being on treatment).^11^ Caregiver health literacy remains understudied in pediatric oncology^12^ and may contribute to COVID‐19 vaccine uptake among young CCS. Health literacy refers to people's capability to acquire and interpret health information to maintain and further improve health.^13^, ^14^ The relationship between health literacy and vaccination acceptance is unclear, with different studies showing contrasting results.^15^ Recent studies addressing the relationship between health literacy and COVID‐19 vaccination found lower health literacy is associated with lower vaccine uptake^16^, ^17^, ^18^, ^19^ and results in higher vaccination hesitancy by mediating the effect of distrust^20^ or with the moderating role of stress.^21^ Data on caregivers' decision to vaccinate young CCS against COVID‐19 is scarce, and the current study seeks to fill this knowledge gap. Caregivers of CCS often have more experience with medical information acquisition and decision‐making, which may increase their overall health literacy and ultimately affect their information behavior, critical thinking, and health decision‐making.

**FIGURE 1:**
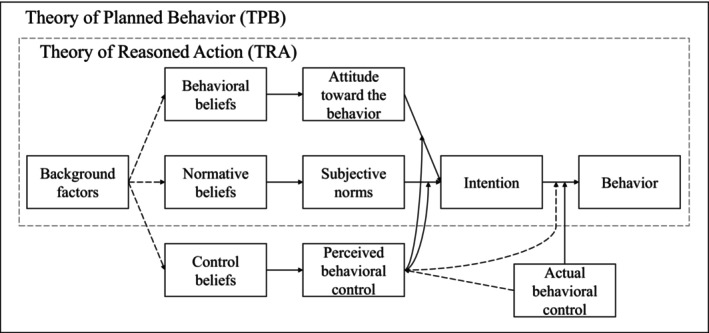
The Theory of Reasoned Action and the Theory of Planned Behavior.

According to the Theory of Reasoned Action (TRA)^22^ and the Theory of Planned Behavior (TPB),^23^ the intention to perform behaviors best predicts actual behavioral performance (Figure 1). The TRA suggests that intention is formed by attitudes (i.e., subjective evaluation of a specific behavior) and subjective norms (i.e., perception of the social support regarding a specific behavior), which result from behavioral and normative beliefs, respectively.^22^ The TPB extends the TRA^24^ by adding the concept of perceived behavioral control (i.e., perception of the ease or difficulty of performing a specific behavior), which is formed by control beliefs.^23^, ^25^ While attitudes and subjective norms are direct predictors of intention, according to original and more recent formulations of the TPB, perceived behavioral control mediates attitudes and subjective norms.^26^ That is, the direction and degree to which attitudes and subjective norms contribute to the intention to perform a behavior are determined by perceived behavioral control. Furthermore, background variables (e.g., sociodemographic characteristics) only indirectly influence intention through attitudes, subjective norms, and perceived behavioral control. The TPB has been applied to study different health behaviors, including COVID‐19 vaccination decisions among adults^27^, ^28^, ^29^ and parents for their children in the general population.^29^, ^30^, ^31^, ^32^ We aimed to use the TPB to identify antecedents contributing to caregivers' decision to vaccinate young CCS against COVID‐19, including health literacy related to COVID‐19 aspects (referred to as “COVID‐19 health literacy” hereafter) and frequency of COVID‐19 information‐seeking.

### 1.1 Hypotheses

To explore the applicability of the TPB to explain COVID‐19 vaccination intentions (i.e., intention) among caregivers of CCS, we tested the following hypotheses:

1. Higher intention is associated with higher attitudes (H1), subjective norms (H2), and perceived behavioral control (H3) (i.e., positive correlations).
2. Perceived behavioral control strengthens the relationships between intention and attitudes (H4a) and between intention and subjective norms (H4b) by moderating them.
3. Higher intention is related to COVID‐19 vaccination (i.e., behavior; H5).

For all significant antecedents of intention (H1‐H4b), we further expected that higher caregiver COVID‐19 health literacy (H6) and frequency of COVID‐19 information‐seeking (H7) are indirectly related to higher intention.

## 2 METHODS

### 2.1 Participants

This cross‐sectional study is part of a larger clinical trial of Cancer SurvivorLink™ (www.cancersurvivorlink.org; NCT03543852), which includes CCS who are at least 2 years off therapy, English‐ or Spanish‐speaking, without any terminal diagnosis, and engaged in survivor care at one of 12 COG clinics across the Midwest (*n* = 4), Northeast (*n* = 4), West (*n* = 3), and South (*n* = 1) regions of the U.S.^33^ Eligible participants were caregivers of CCS who were aged 5–17 years at survey completion and engaged in long‐term follow‐up care in a cancer survivor clinic. Caregivers who were not fluent in either English or Spanish were excluded.

### 2.2 Procedure

Eligible caregivers enrolled in the clinical trial were invited to complete an ancillary online survey on COVID‐19, with up to three email reminders. Data were collected and managed using secure, web‐based REDCap (Research Electronic Data Capture) tools hosted at Emory University.^34^, ^35^ Participants provided online consent before completing the survey and received a $20 gift card. Data collection was conducted between May and June 2022. At the time of data collection, the U.S. Food and Drug Administration (FDA) emergency approval of the Pfizer‐BioNTech COVID‐19 vaccine against COVID‐19 among children five through 11 years of age had been in place for 6 months.^36^ Considering that COVID‐19 vaccines were previously available for older age groups,^37^, ^38^ at the time of data collection, all CCS have been eligible for a COVID‐19 vaccine for at least 6 months. The study received ethical approvals from the Western Institutional Review Board (20200913) and Emory Institutional Review Board (00101506).

### 2.3 Measurements

Item wording and descriptive statistics of all Likert‐type questions are available in Appendix S1. Attitudes (3 items, e.g., “I believe vaccines can help control the spread of COVID‐19.”), subjective norms (2 items, e.g., “Now that the COVID‐19 vaccine is available in my community, my decision of whether or not to get my child vaccinated would depend on: Recommendation from my family doctor”), and perceived behavioral control (1 item, i.e., “Now that the COVID‐19 vaccine is available in my community, my decision of whether or not to get my child vaccinated would depend on: How easy it is to get the vaccine (e.g., available out‐of‐hours or in pharmacies)”) were measured on 7‐point Likert‐type scales. All items were extracted from the survey tool and guidance developed by the World Health Organization (WHO).^39^ When necessary, question‐wording was adapted to better fit the context of the current study. Composite scores measuring attitudes (range: 3–21) and subjective norms (range: 2–14) were generated, with higher scores indicating higher (or more positive) levels of the respective constructs. Perceived behavioral control was used in further analyses as a single item (range: 1–7). Internal consistency of the composite scores of attitudes (Cronbach's alpha (*α*) = 0.750) and subjective norms (*α* = 0.908) was good to very good. Intention (measured on a 5‐point Likert‐type scale) and behavior (yes/no) were assessed using one item each. To facilitate the analysis, we generated a binary variable of intention with labels “no or undecided” (strongly disagree, disagree, or undecided) and “yes” (agree or strongly agree).

COVID‐19 health literacy (9 items) and frequency of COVID‐19 information‐seeking (1 item) were assessed as recommended in the survey tool and guidance of the WHO.^39^ Answer options were given on 5‐point (COVID‐19 health literacy) and 7‐point (frequency of COVID‐19 information‐seeking) Likert‐type scales. We generated a composite variable for COVID‐19 health literacy by summing individual item scores (range: 9–45, *α* = 0.910).

Information on COVID‐19 included caregivers' vaccination status (yes/no), their perceptions about the risk for severe COVID‐19 symptoms if the CCS gets infected (yes/no), having received a personal recommendation from a healthcare professional (HCP) to have the CCS vaccinated (yes/no), and information about the COVID‐19 history of the CCS (no diagnosis, suspected diagnosis, or confirmed diagnosis).

The baseline survey of the larger trial assessed additional sociodemographic data about the caregiver (relationship to the child, race/ethnicity, marital status, education, household income, and employment in healthcare within the household) and CCS (sex, age, original cancer diagnosis, time since diagnosis, and time since end of treatment).

### 2.4 Analysis

Descriptive statistics, including means (*M*), standard deviations (S.D.), skewnesses, kurtoses, and frequencies, were computed for all dependent and independent variables. Unadjusted linear regressions were used to identify significant background variables associated with attitudes, subjective norms, and perceived behavioral control. When the categories of the independent variables were very small (<20 observations), we combined them to form larger categories to reduce the risk of over‐fitting the model. The results of unadjusted linear regressions are available in Appendix S2. Stata (version 17)^40^ was used to run descriptive statistics and unadjusted linear regressions.

Structural equation modeling was conducted in Mplus 8.8.^41^ Model building was sequential based on the hypotheses from regression models to the full path model. Four models were estimated to test our hypotheses. Model 1 examined the direct relationship between caregivers' vaccination intentions and attitudes, subjective norms, and perceived behavioral control. Model 2 tested the indirect relationships between attitudes and intention, and subjective norms and intention, through the moderation of perceived behavioral control. Model 3 tested the full TPB model. Model 4 tested all the previously identified significant paths and incorporated relevant background variables. All models used full information maximum likelihood assuming data missing at random. Due to the inclusion of noncontinuous endogenous variables, the weighted least squares mean and variance adjusted (WLSMV) estimator was used.^41^ Model fit was assessed using Root Mean Square Error of Approximation (RMSEA), Comparative Fit Index (CFI), Tucker‐Lewis Index (TLI), Standardized Root Mean Square Residual (SRMR), and chi‐square.^42^ Statistical significance for all analyses was set at *p* < 0.05, using a two‐sided test. The data analyzed for this study are available upon request.

## 3 RESULTS

### 3.1 Sample characteristics

Of the 237 caregivers invited to participate, 160 caregivers (87.5% biological mothers) of 160 CCS completed the survey and were included in the analyses (67.5% response rate). Caregiver and CCS demographics are outlined in Tables 1 and 2, respectively. No demographic differences were found between caregivers who participated in this survey and those who did not respond to the invitation (all *p*‐values > 0.20). CCS (44.4% female) were, on average, 12.5 years old at study (S.D. = 2.9; range: 6–17). 70.0% (*n* = 112) of caregivers and 53.8% (*n* = 86) of CCS had received at least one vaccine against COVID‐19. Caregivers reported that most CCS had not received a COVID‐19 diagnosis (*n* = 73; 45.6%), while 30.0% (*n* = 48) were confirmed with COVID‐19, and 18.8% (*n* = 30) were suspected to have had COVID‐19.

**TABLE 1:** Characteristics of caregivers.

Characteristics of caregivers	*n*	%
Relationship to the child		
Biological mother	140	87.5
Biological father	15	9.4
Adoptive mother	2	1.3
Legal guardian	2	1.3
Missing	1	0.6
Race/ethnicity		
White/non‐Hispanic	121	75.6
Hispanic	18	11.3
More than one race	9	5.6
Asian	5	3.1
Black or African American	3	1.9
Missing	4	2.5
Marital status		
Married or living with a partner	126	78.8
Divorced, widowed, or separated	20	12.5
Single (or never married)	13	8.1
Missing	1	0.6
Education (highest completed)		
Less than high school	5	3.1
High school diploma or equivalent	24	15.0
Some college, but no degree	23	14.4
Associate's degree	33	20.6
College or bachelor's degree	38	23.8
Graduate degree or higher (e.g., master's)	36	22.5
Missing	1	0.6
Household income		
Less than $50,000	29	18.1
$50,000–$99,999	55	34.4
$100,000–$150,000	34	21.3
Over $150,000	31	19.4
Missing	11	6.9
Employment in healthcare within household		
No	96	60.0
Yes	53	33.1
Missing	11	6.9
Vaccination status		
Vaccinated	112	70.0
Not vaccinated	35	21.9
Missing	13	8.1

*Note*: Total percentages might not add up exactly to 100 because of rounding issues.

**TABLE 2:** Characteristics of childhood cancer survivors (CCS).

Characteristics of CCS	*n*	%	*M*	S.D.
Sex				
Female	71	44.4		
Male	89	55.6		
Age at study (years)			12.5	2.9
Vaccination status				
Vaccinated	86	53.8		
Not vaccinated	61	38.1		
Missing	13	8.1		
Original cancer diagnosis				
Leukemia	87	54.4		
Neuroblastoma	20	12.5		
Wilms tumor	16	10.0		
Bone & soft tissue sarcoma	14	8.8		
Lymphoma	7	4.4		
Other^a^	16	10.0		
Time since diagnosis (years)^b^			9.7	3.3
Time since end of treatment (years)^b^			8.0	3.5
COVID‐19 history				
No COVID‐19 diagnosis	73	45.6		
Suspected COVID‐19 diagnosis	30	18.8		
Confirmed COVID‐19 diagnosis	48	30.0		
Missing	9	5.6		

*Note*: Total percentages might not add up exactly to 100 because of rounding issues.

Abbreviations: CNS, central nervous system; *M*, mean; S.D., standard deviation.

^a^ Includes: Hepatoblastoma (*n* = 4); Retinoblastoma (*n* = 4); Langerhans cell histiocytosis (*n* = 4); Germ cell tumor (*n* = 2); Pleuropulmonary blastoma (*n* = 1); CNS tumor (*n* = 1).

^b^ Missing: *n* = 1.

More than two‐thirds of respondents reported receiving personal recommendations from a HCP to vaccinate the CCS against COVID‐19 (*n* = 108; 67.5%), and 48.8% (*n* = 78) perceived their child's cancer history increases their risk for more severe COVID‐19 symptoms. More than one‐third of respondents (37.5%) reported either strong disagreement (*n* = 38), disagreement (*n* = 4), or indecision (*n* = 18) about future COVID‐19 vaccination for the CCS, while 55.0% reported strong agreement (*n* = 82) or agreement (*n* = 6). Twelve respondents (7.5%) did not report their preference. Caregivers of CCS aged 5–11 years old showed lower agreement about future vaccination (*n* = 28; 46.7%), whereas caregivers of CCS aged 12–17 years old expressed higher agreement (*n* = 60; 60.0%). Caregivers reported relatively high COVID‐19 health literacy (*M* = 34.7; S.D. = 6.8; range: 18–45) and moderate COVID‐19 information‐seeking (*M* = 3.1; S.D. = 1.5; range: 1–7).

### 3.2 Structural equation modeling

A graphic representation of the four estimated models is available in Appendix S3, and all model fit data are available in Table 3. In Model 1, attitudes (standardized beta coefficient (*β*) = 0.508; standard error (S.E.) = 0.082; *p*‐value (*p*) < 0.001) and subjective norms (*β* = 0.320; S.E. = 0.065; *p* < 0.001) were both positively associated with intention, supporting H1 and H2. We did not find any direct (*p* = 0.560) association between perceived behavioral control and intention, rejecting H3. In Model 2, both moderations of attitudes (*p* = 0.551) and subjective norms (*p* = 0.582) were not associated with intention, rejecting H4a and H4b. Model 3 showed that caregivers' intention (*β* = 0.864; S.E. = 0.054; *p* < 0.001) was related to the CCS vaccination status, supporting H5. Attitudes (*β* = 0.478; S.E. = 0.151; *p* = 0.001) and subjective norms (*β* = 0.401; S.E. = 0.128; *p* = 0.002) were both directly associated with intention, but perceived behavioral control (*p* = 0.820) and its moderation of attitudes (*p* = 0.618) and subjective norms (*p* = 0.521) all remained not significant. We further observed substantial *R*‐squared (*R* ^2^) values for both endogenous variables (behavior: *R* ^2^ = 0.746; S.E. = 0.092; *p* < 0.001 and intention: *R* ^2^ = 0.590; S.E. = 0.080; *p* < 0.001).

**TABLE 3:** Models fit data.

Fit index	Good fit	Models 1 & 2	Model 3	Model 4
RMSEA	*p* < 0.08 reasonable fit; *p* < 0.05 good fit	N/A as these are regression models (i.e., they are saturated and have 0 degrees of freedom)	*p* < 0.001	*p* < 0.001
CFI	*p* > 0.90 or *p* > 0.95		*p* = 1.000	*p* = 1.000
TLI	*p* > 0.95		*p* = 1.000	*p* = 1.000
SRMR	*p* < 0.08		*p* = 0.169	*p* = 0.036
Chi‐square (model vs. saturated)	*p* > 0.05		*p* = 0.655	*p* = 0.776

Abbreviations: CFI, Comparative Fit Index; RMSEA, Root Mean Square Error of Approximation; SRMR, Standardized Root Mean Square Residual; TLI, Tucker‐Lewis Index.

**FIGURE 2:**
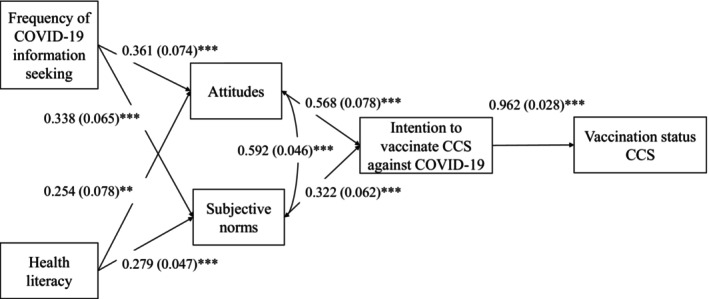
Final full path model (Model 4) with standardized coefficients estimates and standard errors (in brackets). Significance levels: *p < 0.05, **p < 0.01, ***p < 0.001.

In Model 4 (Figure 2), frequency of COVID‐19 information‐seeking and COVID‐19 health literacy were both significantly related to attitudes (*β* = 0.361; S.E. = 0.074; *p* < 0.001 and *β* = 0.254; S.E. = 0.078; *p* = 0.001, respectively) and subjective norms (*β* = 0.338; S.E. = 0.065; *p* < 0.001 and *β* = 0.279; S.E. = 0.047; *p* < 0.001, respectively). Attitudes (*β* = 0.568; S.E. = 0.078; *p* < 0.001) and subjective norms (*β* = 0.322; S.E. = 0.062; *p* < 0.001) were associated with intention, which in turn was associated with behavior (*β* = 0.962; S.E. = 0.028; *p* < 0.001). Additionally, frequency of COVID‐19 information‐seeking and COVID‐19 health literacy were both indirectly associated with intention (*β* = 0.313; S.E. = 0.063; *p* < 0.001 and *β* = 0.234; S.E. = 0.059; *p* < 0.001, respectively), supporting H6 and H7, and behavior (*β* = 0.302; S.E. = 0.062; *p* < 0.001 and *β* = 0.225; S.E. = 0.055; *p* < 0.001, respectively). Given the sample size of *n* = 160, we have constrained the number of background variables in the final model to ensure a robust analysis. The results of the unadjusted regressions did not show any statistically significant relationships between attitudes and subjective norms with any cancer‐related variable (original cancer diagnosis, time since diagnosis, and time since the end of treatment). While additional background variables were associated with attitudes and subjective norms in unadjusted regression analyses (Appendix S2), we have chosen to concentrate on the variables of primary interest to this study, namely frequency of COVID‐19 information‐seeking and COVID‐19 health literacy. Model 4 further showed substantial *R*‐squared values for the following endogenous variables: behavior (*R* ^2^ = 0.926; S.E. = 0.053; *p* < 0.001), intention (*R* ^2^ = 0.679; S.E. = 0.090; *p* < 0.001), attitudes (*R* ^2^ = 0.252; S.E. = 0.046; *p* < 0.001), and subjective norms (*R* ^2^ = 0.251; S.E. = 0.048; *p* < 0.001). Although both models showed an excellent fit to the data based on CFI, TLI, and RMSEA fit indexes, Model 4 seems to be a better fitting model than Model 3 based on the improvement in the SRMR, *R*‐squared, and chi‐squared *p*‐values.

## 4 DISCUSSION

Improving COVID‐19 vaccination rates among CCS may help reduce their risk for a severe disease course and other complications later in life. Despite national recommendations and the widespread availability of vaccines against COVID‐19, only half of the CCS in our sample had received a COVID‐19 vaccine. Applying the TPB, we found that the COVID‐19 vaccination status of CCS was strongly associated with caregivers' vaccine intentions, with intentions being shaped by caregivers' attitudes, subjective norms (i.e., perceived support), COVID‐19 health literacy, and frequency of COVID‐19 information‐seeking.

According to a nationally representative study conducted in October–November 2021, 54.0% of parents of children aged 5–11 years old and 69.7% of parents of children aged 12–17 years old indicated a likelihood of vaccinating or having already vaccinated their child against COVID‐19.^43^ The current study suggests that acceptance rates among parents of CCS might be slightly lower, even though CCS may be at higher risk for COVID‐19 infection, a severe course of the disease, long‐lasting symptoms, and further complications. Compared to previous research conducted among caregivers of minor CCS in the U.S.,^10^, ^11^ our study identified more unfavorable and less neutral intentions toward COVID‐19 vaccines for CCS. Further, our results showed that most caregivers were either strongly favorable or unfavorable in their intentions toward COVID‐19 vaccines for their CCS, with very few participants reporting moderate views or indecision. Previous research on CCS caregivers' COVID‐19 vaccine acceptancy was conducted in early 2021,^10^, ^11^ when the Pfizer‐BioNTech COVID‐19 vaccine was authorized for emergency use in individuals aged 16 and older, and no vaccines were available for younger children.^44^ As vaccines became available for children worldwide, online information promoting mistrust in the vaccine and misinformation may have raised concerns among caregivers.^45^ Additionally, the increasing polarization toward COVID‐19 vaccines associated with political beliefs may have also contributed to observed differences in intentions.^46^

Our results showed that attitudes and perceived support were associated with caregivers' vaccine intentions. Perceived behavioral control was not directly associated with intention or moderate the relationships between attitudes or subjective norms and intention. The TRA applies to straightforward behaviors over which individuals have reasonable control.^24^ Given that COVID‐19 vaccination is relatively easy, accessible, and under people's control, perceived behavioral control may have less influence in explaining intention. Previous studies that used the TPB to examine parents' COVID‐19 vaccination intentions for their children have produced contrasting results in the general population. Two studies were conducted in China^30^ and Hong Kong^32^ before vaccines were authorized for children and at the time the first vaccines were becoming available for children, respectively. Both studies found all constructs, attitudes, subjective norms, and perceived behavioral control to be related to intention. Conversely, a study conducted in China after the authorization for COVID‐19 vaccines among children showed similar results to ours and did not find perceived behavioral control to be indicative of parents' intentions.^31^ Based on our findings and the previous research, we can conclude that perceived behavioral control is important for caregivers when vaccines are not accessible for their child. However, when vaccines become accessible, attitudes and perceived support from HCP may be more critical in shaping vaccine intentions and behavior.

Misconceptions and safety concerns regarding COVID‐19 vaccines may contribute to vaccine hesitancy,^47^ especially among individuals with lower health literacy or those who are underinformed and more vulnerable to misinformation. Our study found that more frequent COVID‐19 information‐seeking and higher COVID‐19 health literacy were indirectly associated with positive intentions for vaccination. These findings are consistent with previous research on COVID‐19 vaccination^16^, ^17^, ^18^, ^19^ and highlight the importance of caregivers' health literacy in understanding the risks and benefits of vaccination and making informed decisions for their children. Given the extensive media coverage and conflicting information surrounding COVID‐19 vaccines, individuals with lower COVID‐19 health literacy may have difficulty distinguishing accurate information from myths or misinformation, leading to lower vaccination intentions. Conversely, actively seeking and evaluating reliable sources of information may increase positive beliefs toward vaccination and intention to vaccinate.^48^ Thus, promoting clear communication about COVID‐19 vaccines, especially tailored to the childhood cancer context, encouraging caregivers to review reputable sources of accurate information, and monitoring and counteracting disinformation around vaccines, especially in the pediatric setting, may help address vaccine hesitancy and increase positive beliefs about COVID‐19 vaccination among caregivers of CCS.

### 4.1 Strengths and limitations

When interpreting our results, it is important to consider the limitations related to our sample. Firstly, only caregivers of CCS who were engaged in long‐term follow‐up care at a cancer survivor clinic were eligible for participation, which might limit the generalizability of our findings. Because these caregivers were engaged in long‐term follow‐up care, they may have more concerns about the health of the CCS and, therefore, more favorable and willing to have them vaccinated against COVID‐19. Secondly, while we could recruit sufficient participants, we acknowledge that our sample size was still relatively limited for SEM analyses. To address this limitation, we reduced the complexity of our model, which nevertheless successfully explained COVID‐19 vaccination intentions for CCS among caregivers. Thirdly, our sample was predominantly composed of white/non‐Hispanic biological mothers and showed higher educational attainment and income than the U.S. population, which may limit the generalizability of our findings to other subpopulations of CCS. We recommend that future research includes more male caregivers and specifically seeks to understand vaccine intentions among Black and Hispanic families of CCS, as well as among families with lower socioeconomic status, given observed racial/ethnic^49^ and socioeconomic^50^ disparities in rates of COVID‐19 infection and vaccine uptake. Lastly, our study was cross‐sectional, and we could not assess vaccination intention and behavior at different times. However, we expect that current vaccination status and future vaccination behavior are related, given that caregivers had sufficient time to decide whether to vaccinate the CCS.

Several strengths suggest that this study's results are reliable and informative for understanding COVID‐19 vaccination intentions for CCS among caregivers. Firstly, the data were collected from families recruited from 12 clinics across the U.S., increasing the geographic reach and generalizability of the findings. Secondly, the analysis showed an outstanding model fit to the data, demonstrating the statistical methods' robustness. Furthermore, the study was theory‐driven. We used well‐established health behavior theories to guide measurement and interpret our findings. This theoretical foundation enhances the rigor and coherence of the study and supports the validity of our results.

### 4.2 Conclusion

Attitudes, perceived support, frequency of COVID‐19 information‐seeking, and COVID‐19 health literacy shape caregivers' COVID‐19 vaccination intentions and behavior for their minor CCS. Our results may have several important implications for future research and practice in pediatric vaccinations, such as MMR or HPV vaccines, and vaccines against future pandemics, where similar attitudes, norms, and control perceptions may apply. Further research is needed to explore the relationship between these psychological factors and understand how they influence vaccine acceptance and uptake in other scenarios. The TRA was a valuable and comprehensive framework for studying CCS caregivers' COVID‐19 vaccine intentions, overperforming the more complex TPB. Furthermore, our results suggest that communication persuading caregivers to vaccinate their CCS should not focus on the easiness of receiving a vaccine once it is widely accessible. Frequency of COVID‐19 information‐seeking and COVID‐19 health literacy were important indicators for explaining caregivers' intention toward COVID‐19 vaccination. These are modifiable variables to which public health efforts should be dedicated to increase vaccination intentions and ultimately improve vaccine uptake among young CCS.

## AUTHOR CONTRIBUTIONS

**Anica Ilic:** Conceptualization (equal); data curation (equal); formal analysis (equal); funding acquisition (equal); methodology (equal); project administration (lead); visualization (lead); writing – original draft (lead); writing – review and editing (equal). **Regine Haardoerfer:** Conceptualization (equal); data curation (equal); formal analysis (equal); methodology (equal); writing – review and editing (equal). **Gisela Michel:** Writing – review and editing (equal). **Cam Escoffery:** Conceptualization (equal); funding acquisition (equal); methodology (equal); writing – review and editing (equal). **Ann C. Mertens:** Conceptualization (equal); funding acquisition (equal); methodology (equal); writing – review and editing (equal). **Jordan Gilleland Marchak:** Conceptualization (equal); methodology (equal); writing – review and editing (equal).

## CONFLICT OF INTEREST STATEMENT

The authors declare no conflicts of interest.

## Supporting information


Appendix S1.

Appendix S2.

Appendix S3.

## Data Availability

The data analyzed for this study are available upon request.
