# Supplementary material for: Understanding caregivers' decision to vaccinate childhood cancer survivors against COVID‐19
Source: Cancer Med. 2023 Nov 8;12(23):21354–63. doi: 10.1002/cam4.6675 (PMC10726781; doi:10.1002/cam4.6675)
Supplement: Supplementary file 1 — Appendix S1. [file CAM4-12-21354-s003.docx]

**Appendix S1. Item wording and descriptive statistics of Likert-type questions.**

| Construct | Item | Question | Original answer options | n | Mean | S.D. | Skewness | Kurtosis |
| --- | --- | --- | --- | --- | --- | --- | --- | --- |
| Attitudes | 1 | I believe vaccines can help control the spread of COVID-19. | 1 Strongly disagree  7 Strongly agree | 152 | 4.83 | 2.19 | -0.50 | 1.78 |
|  | 2* | When everybody is vaccinated against COVID-19, I don't have to. | 1 Strongly disagree  7 Strongly agree | 150 | 2.13 | 1.63 | 1.43 | 4.23 |
|  | 3* | If I knew my child had COVID-19, I would not have him/her get the vaccine. | 1 Strongly disagree  7 Strongly agree | 147 | 3.03 | 2.35 | 0.62 | 1.83 |
| Subjective norms | 1 | Now that the COVID-19 vaccine is available in my community, my decision of whether or not to get my child vaccinated would depend on:  Recommendation from my family doctor | 1 Not at all  7 Very much so | 156 | 4.85 | 2.21 | -0.61 | 1.97 |
|  | 2 | Now that the COVID-19 vaccine is available in my community, my decision of whether or not to get my child vaccinated would depend on:  Recommendation of the federal health agencies (i.e., CDC or Centers for Disease Control and Prevention) | 1 Not at all  7 Very much so | 158 | 4.37 | 2.31 | -0.34 | 1.60 |
| Perceived behavioral control | 1 | Now that the COVID-19 vaccine is available in my community, my decision of whether or not to get my child vaccinated would depend on:  How easy it is to get the vaccine (e.g., available out-of-hours or in pharmacies) | 1 Not at all  7 Very much so | 155 | 3.88 | 2.20 | 0.06 | 1.64 |
| Intention | 1 | I will have my child (survivor) vaccinated. | 1 Strongly disagree  5 Strongly agree | 148 | 3.61 | 1.72 | -0.63 | 1.64 |
| Frequency of COVID-19 information-seeking | 1 | How often do you seek information about COVID-19? | 1 Never  7 Several times a day | 151 | 3.13 | 1.46 | 0.26 | 2.61 |

(Continued on next page)

| Construct | Item | Question | Original answer options | n | Mean | S.D. | Skewness | Kurtosis |
| --- | --- | --- | --- | --- | --- | --- | --- | --- |
| Health literacy | 1 | How easy or difficult would you say it is to…: …find the information you need related to COVID-19? | 1 Very difficult  5 Very easy | 149 | 3.99 | 0.93 | -0.43 | 2.34 |
|  | 2 | How easy or difficult would you say it is to…: …understand information about what to do if you think you have COVID-19? | 1 Very difficult  5 Very easy | 149 | 4.16 | 0.88 | -0.56 | 2.11 |
|  | 3 | How easy or difficult would you say it is to…: …judge if the information about COVID-19 in the media is reliable? | 1 Very difficult  5 Very easy | 149 | 3.07 | 1.26 | -0.09 | 2.15 |
|  | 4 | How easy or difficult would you say it is to…: …understand restrictions and recommendations of authorities regarding COVID-19? | 1 Very difficult  5 Very easy | 149 | 3.74 | 1.09 | -0.56 | 2.62 |
|  | 5 | How easy or difficult would you say it is to…: …follow the recommendations on how to protect yourself from COVID-19? | 1 Very difficult  5 Very easy | 149 | 4.01 | 0.92 | -0.64 | 3.04 |
|  | 6 | How easy or difficult would you say it is to…: …understand recommendations about when to stay at home from work/school, and when not to? | 1 Very difficult  5 Very easy | 149 | 3.95 | 1.02 | -0.62 | 2.42 |
|  | 7 | How easy or difficult would you say it is to…: …follow recommendations about when to stay at home from work/school, and when not to? | 1 Very difficult  5 Very easy | 145 | 3.80 | 1.05 | -0.53 | 2.38 |
|  | 8 | How easy or difficult would you say it is to…: …understand recommendations about when to engage in social activities, and when not to? | 1 Very difficult  5 Very easy | 148 | 3.96 | 0.91 | -0.35 | 2.10 |
|  | 9 | How easy or difficult would you say it is to…: …follow recommendations about when to engage in social activities, and when not to? | 1 Very difficult  5 Very easy | 148 | 3.78 | 1.01 | -0.40 | 2.41 |

Notes:

* The following table shows scores according to the original answer options. Scores have been reverse-coded for further analyses so that higher values indicate more favorable attitudes.

Abbreviations: CCS, childhood cancer survivor; HCP, healthcare professional; n, number of participants; S.D., standard deviation.
