# Supplementary material for: Understanding caregivers' decision to vaccinate childhood cancer survivors against COVID‐19
Source: Cancer Med. 2023 Nov 8;12(23):21354–63. doi: 10.1002/cam4.6675 (PMC10726781; doi:10.1002/cam4.6675)
Supplement: Supplementary file 2 — Appendix S2. [file CAM4-12-21354-s002.docx]

**Appendix S2. Results from unadjusted linear regressions.**

|  |  | Attitudes | | | | Subjective norms | | | | Perceived behavioral control | | | |
| --- | --- | --- | --- | --- | --- | --- | --- | --- | --- | --- | --- | --- | --- |
|  | n^a^ | Coef. | 95% CI | | p-value | Coef. | 95% CI | | p-value | Coef. | 95% CI | | p-value |
| Frequency of COVID-19 information-seeking | 151 | **1.56** | **1.05** | **2.07** | **<0.001** | **1.23** | **0.80** | **1.67** | **<0.001** | **0.46** | **0.23** | **0.69** | **<0.001** |
| Health literacy | 143 | **0.28** | **0.17** | **0.40** | **<0.001** | **0.25** | **0.15** | **0.35** | **<0.001** | **0.08** | **0.03** | **0.13** | **0.003** |
| Caregiver’s vaccination status | 147 |  |  |  | **<0.001** |  |  |  | **<0.001** |  |  |  | **<0.001** |
| No (Ref.) | 35 | **0** |  |  |  | **0** |  |  |  | **0** |  |  |  |
| Yes | 112 | **6.09** | **4.41** | **7.76** |  | **4.65** | **3.18** | **6.13** |  | **1.92** | **1.15** | **2.69** |  |
| Perceived risk for severe COVID-19 symptoms | 151 |  |  |  | **<0.001** |  |  |  | **<0.001** |  |  |  | **0.003** |
| No (Ref.) | 73 | **0** |  |  |  | **0** |  |  |  | **0** |  |  |  |
| Yes | 78 | **3.87** | **2.34** | **5.41** |  | **2.44** | **1.10** | **3.78** |  | **1.03** | **0.34** | **1.71** |  |
| Personal recommendation from HCP | 141 |  |  |  | 0.054 |  |  |  | **0.003** |  |  |  | 0.142 |
| No (Ref.) | 33 | 0 |  |  |  | **0** |  |  |  | 0 |  |  |  |
| Yes | 108 | 1.95 | -0.03 | 3.94 |  | **2.50** | **0.86** | **4.14** |  | 0.63 | -0.21 | 1.47 |  |
| Race/Ethnicity | 156 |  |  |  | **0.001** |  |  |  | 0.051 |  |  |  | **0.020** |
| White/non-Hispanic (Ref.) | 121 | **0** |  |  |  | 0 |  |  |  | **0** |  |  |  |
| Other | 35 | **3.33** | **1.33** | **5.33** |  | 1.64 | -0.01 | 3.29 |  | **1.01** | **0.16** | **1.85** |  |
| Education | 159 |  |  |  | 0.261 |  |  |  | 0.102 |  |  |  | 0.471 |
| High school diploma or less (Ref.) | 29 | 0 |  |  |  | 0 |  |  |  | 0 |  |  |  |
| Some college, but no degree | 23 | -1.15 | -4.16 | 1.86 |  | -0.69 | -3.09 | 1.72 |  | 0.27 | -0.99 | 1.53 |  |
| Associate's degree | 33 | -1.03 | -3.76 | 1.69 |  | -1.68 | -3.87 | 0.52 |  | -0.36 | -1.50 | 0.79 |  |
| College or bachelor's degree | 38 | -0.47 | -3.12 | 2.18 |  | -0.53 | -2.67 | 1.61 |  | -0.49 | -1.60 | 0.62 |  |
| Graduate degree or higher | 36 | 1.46 | -1.25 | 4.17 |  | 1.14 | -1.02 | 3.29 |  | 0.32 | -0.80 | 1.44 |  |
| Household income | 149 |  |  |  | 0.161 |  |  |  | 0.384 |  |  |  | 0.764 |
| Less than 50,000 (Ref.) | 29 | 0 |  |  |  | 0 |  |  |  | 0 |  |  |  |
| 50,000-99,999 | 55 | 1.96 | -0.44 | 4.36 |  | 0.31 | -1.70 | 2.32 |  | 0.28 | -0.75 | 1.31 |  |
| 100,000-150,000 | 34 | 2.97 | 0.38 | 5.57 |  | 1.54 | -0.65 | 3.74 |  | -0.03 | -1.16 | 1.10 |  |
| Over 150,000 | 31 | 1.73 | -0.95 | 4.41 |  | -0.14 | -2.38 | 2.11 |  | -0.24 | -1.39 | 0.91 |  |

(Continued on next page)

|  |  | Attitudes | | | | Subjective norms | | | | | Perceived behavioral control | | | | | |
| --- | --- | --- | --- | --- | --- | --- | --- | --- | --- | --- | --- | --- | --- | --- | --- | --- |
|  | n^a^ | Coef. | 95% CI | | p-value | Coef. | | 95% CI | | p-value | Coef. | | 95% CI | | | p-value |
| Employment in healthcare within the household | 149 |  |  |  | 0.450 |  |  | |  | 0.790 |  |  | |  | 0.767 | |
| No (Ref.) | 96 | 0 |  |  |  | 0 |  | |  |  | 0 |  | |  |  | |
| Yes | 53 | -0.67 | -2.41 | 1.07 |  | -0.20 | -1.67 | | 1.27 |  | 0.11 | -0.62 | | 0.84 |  | |
| Age CCS | 160 | 0.10 | -0.19 | 0.39 | 0.494 | 0.13 | -0.11 | | 0.37 | 0.302 | 0.03 | -0.10 | | 0.15 | 0.669 | |
| Time since end of treatment | 159 | 0.13 | -0.10 | 0.37 | 0.265 | 0.17 | -0.03 | | 0.36 | 0.099 | 0.00 | -0.10 | | 0.10 | 0.937 | |
| COVID-19 history of CCS | 151 |  |  |  | 0.524 |  |  | |  | 0.300 |  |  | |  | 0.084 | |
| No diagnosis (Ref.) | 73 | 0 |  |  |  | 0 |  | |  |  | 0 |  | |  |  | |
| Suspected diagnosis | 30 | -1.17 | -3.37 | 1.04 |  | 0.06 | -1.79 | | 1.92 |  | 0.23 | -0.69 | | 1.15 |  | |
| Confirmed diagnosis | 48 | -0.76 | -2.65 | 1.13 |  | -1.17 | -2.76 | | 0.43 |  | -0.76 | -1.55 | | 0.03 |  | |

Notes: Statistically significant variables at p<0.05 are highlighted in bold.

^a^ n indicates the number of participants who selected each answer option. For each variable, the difference between the total respondents and 160 is the number of missing values.

Abbreviations: CCS, childhood cancer survivor; CI, confidence interval; HCP, healthcare professional; Ref.; reference category.
