# Supplementary material for: Understanding caregivers' decision to vaccinate childhood cancer survivors against COVID‐19
Source: Cancer Med. 2023 Nov 8;12(23):21354–63. doi: 10.1002/cam4.6675 (PMC10726781; doi:10.1002/cam4.6675)
Supplement: Supplementary file 3 — Appendix S3. [file CAM4-12-21354-s001.docx]

**
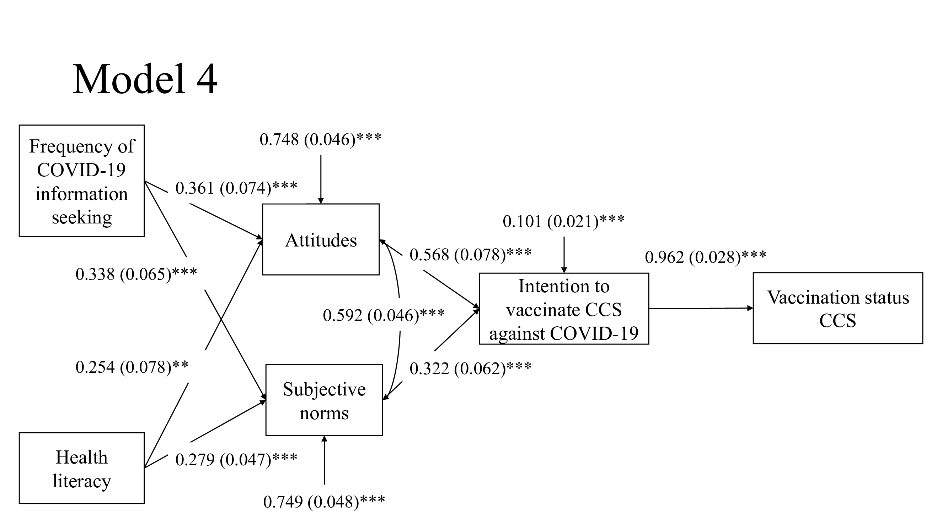

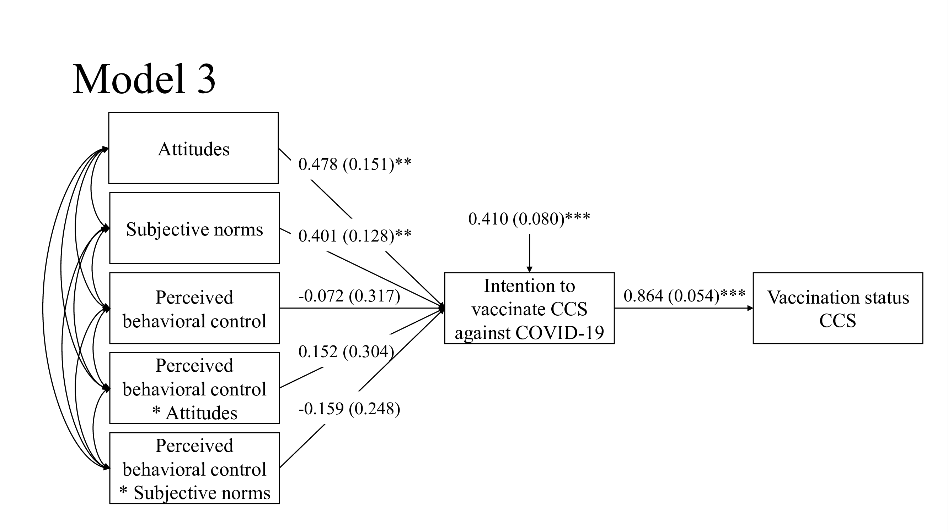

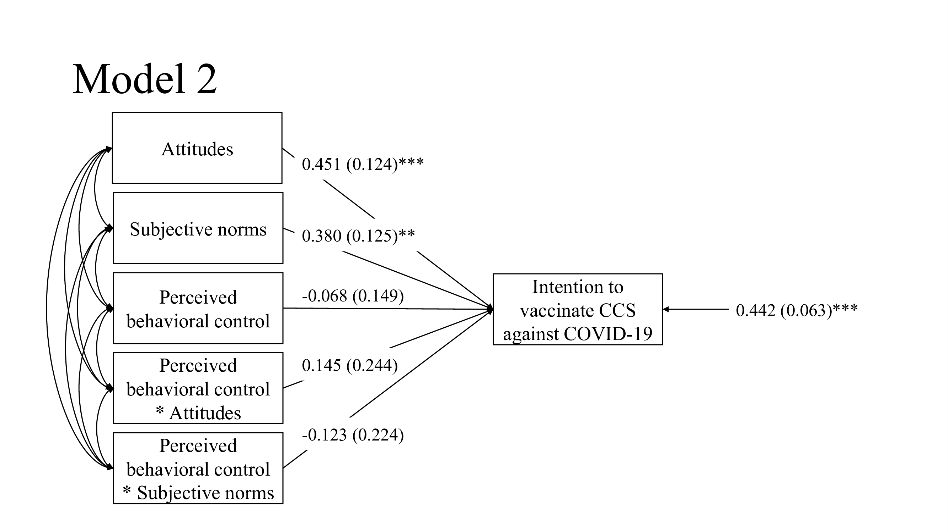

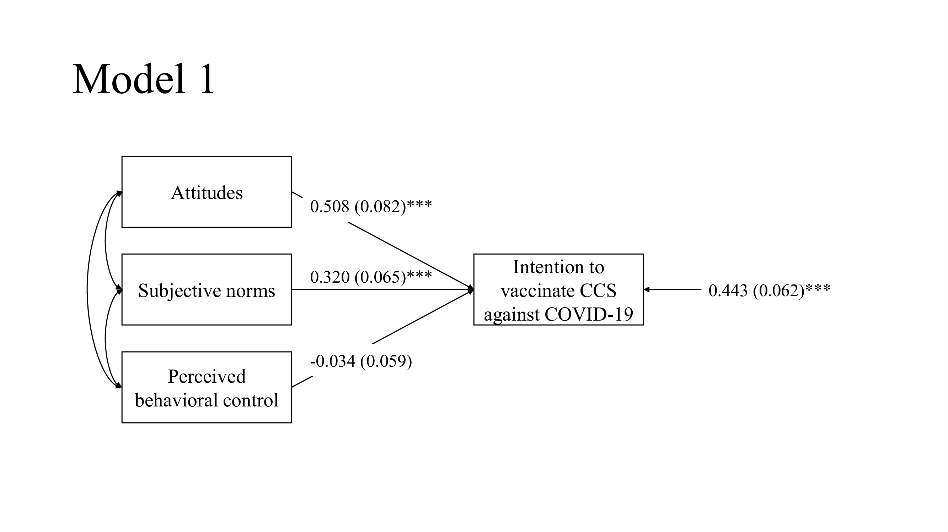
Appendix S3. Representation of the tested models.**

Notes: Standardized beta coefficients and standard errors (in brackets). Significance levels: *p<0.05, **p<0.01, ***p<0.001.

Abbreviations: CCS, childhood cancer survivor; COVID-19, Coronavirus disease 2019.
